# Influence of The Segregation Phenomenon on Structural Efficiency of Lightweight Aggregate Concretes

**DOI:** 10.3390/ma13245754

**Published:** 2020-12-16

**Authors:** Afonso Miguel Solak, Antonio José Tenza-Abril, Victoria Eugenia García-Vera

**Affiliations:** 1 Department of Civil Engineering, University of Alicante, 03080 Alicante, Spain; afonsosolak@gmail.com; 2 CYPE Ingenieros S.A., 03003 Alicante, Spain; 3 Departamento de Arquitectura y Tecnología de la Edificación, Universidad Politécnica de Cartagena, 30203 Murcia, Spain; victoria.eugenia@upct.es

**Keywords:** Concrete segregation, lightweight aggregate concrete, compressive strength, ANOVA, segregation index

## Abstract

Lightweight aggregate concretes (LWAC) are versatile and interesting materials for projects that require greater structural efficiency. Due to the difference that exists between the densities of the materials used in these types of concrete, during transport and mainly compaction, their aggregates tend to separate from the mortar matrix, floating towards the surface, a phenomenon called segregation. Segregation in LWAC can affect its durability properties, its density, and directly affect its structural efficiency. In this work, different concrete densities (1700 kg/m^3^ and 1900 kg/m^3^) manufactured with different dosages (two different lightweight aggregates) and compaction methods (one or two layers) were analyzed to verify the impact of segregation on its structural efficiency. For this purpose, the segregation index of the LWAC was obtained by means of the image analysis technique. In addition, to obtain their structural efficiency, the density and compressive strength were obtained at different heights of the tested specimens. The results show the vibration of the samples in two layers leads to a more efficient elimination of trapped air, a reduction in the risk of segregation, and better structural efficiency.

## 1. Introduction

The ability of a concrete mixture to maintain the homogeneous distribution of all its components is key to success in the application of concrete [1]. The segregation of concrete components is a complex phenomenon and its cause can come from different reasons [2,3]. Concrete compacted with the same level of energy showed different levels of segregation depending on how the compaction was performed [4]. The results of Navarrete and Lopez [5] indicate that the size and shape of the aggregates are other factors that can affect the occurrence of segregation. Previous studies of Solak et al. [6] showed that the combined effect of reducing the concrete density and consistency contributed to the segregation-resistance of the concrete.

The segregation and bleeding of fresh mixture has a dramatic negative impact on the mechanical properties [7,8,9] of hardened concrete as wells as on its durability properties [10]. Traditionally, researches that involve the segregation of concrete were based on visual observation. However, recent research shows that is not always as clear [2,11]. The heterogeneity of concrete can be usually reflected by the volume fraction variation of coarse aggregates along depth and it often arises from gravity [12] induced particle migration.

The inclusion of lightweight aggregates (LWA) in concrete in certain situations can affect its mechanical properties [13], its capillary absorption [14], its oxygen permeability [15], its porosity, and its fluid transport properties [16]. According to studies published by Bogas and Gomes [17], the influence of LWA on LWAC strength increases when LWAs density decreases, when mortar resistance increases and when concrete ages. Some studies have highlighted the importance of analyzing the volume fraction of lightweight aggregates in LWAC considering that variations in dosage can lead to important variations in the LWAC mechanical properties [18,19]. In normal weight concrete, the aggregate strength is not a factor affecting concrete strength because the aggregate is much stronger than the matrix and the transition zone [20]. However, in LWAC, the constituent LWA may have a lower strength and elastic modulus than the mortar matrix, and thus the properties of the LWA are the most important determinants of the properties of the resulting concrete [21] and usually the failure of concrete then is controlled by aggregate fracture [19].

Structural efficiency can be defined in many ways [22] but is usually the value of the load supported by material to its weight. Several researches have used this term for analyzing the performance of different materials [23,24]. Considering that one of the most interesting characteristics of LWAC is its reduced weight, a possible natural tendency for a designer when using this material would be to seek the best density- strength ratio and, as described by Bogas et al. [13], the ratio between compressive strength (f_c_) and density (ρ) of material enables an analysis of its structural efficiency. To what extent should concrete segregation be predicted in an analysis like this? Would this be a phenomenon that would eventually be disregarded in the project, and could it possibly settle differences between the projected material and the material used in construction?

This paper aims to verify the impact of the segregation phenomenon caused by excessive vibration in lightweight aggregate concrete samples on the density, mechanical strength, and structural efficiency of this material. Different types of lightweight aggregate concrete were manufactured and vibrated during their compaction, considering different levels of vibration.

## 2. Materials

Four different LWAC were produced using the Fanjul Method [25], with target densities of 1700 kg/m^3^ (LWAC1 and LWAC3) and 1900 kg/m^3^ (LWAC2 and LWAC4). The method proposed by Fanjul [25] allows the dosage of the aggregates in a concrete composition whose reference is the density of the concrete previously set. Given its pragmatism and efficiency, the method is a powerful working tool, respecting always the weight-volume binomial. The Fanjul method [25] deals with the mixing of the aggregates in the concrete composition. The dosage in cement and water according to the needs of docility, resistance, etc., are considered as known data, either imposed or deducted by other procedures.

Table 1 includes the mix proportions of the concretes produced and presents their manufacturing characteristics. Twenty units of each concrete were manufactured at the same time. Different types of LWA from Arlita Leca (HS and M types), different modes of vibration (one or two layers: LWAC1 and LWAC2 were vibrated in two layers and LWAC3 and LWAC4 were vibrated in one layer) and different theoretical densities were considered. The same water to cement ratio (w/c) of 0.6, was used for all mixtures, resulting in 350 kg/m^3^ of cement and 210 kg/m^3^ of water to produce 1 m^3^ of concrete. CEM I 52.5 R white cement with an absolute density of 3176 kg/m^3^ was used for all the concretes; 2 types of expanded clay were used as coarse lightweight aggregate—its physical properties are listed in Table 2.

The bulk density of the LWAs was obtained according to the procedure described in the standard UNE EN 1097-3 [28]. Particle size of the aggregates was determined according to the UNE EN 933-1 [29]. In addition, the particle density and water absorption were determined by the methodology proposed by Fernández-Fanjul et al. in [27]. According this method, the determination of the particle density was obtained using glycerin to avoid the surface absorption in the aggregates. According this method, the value of the water absorption obtained in the aggregates was more accurate than the values usually obtained according the UNE EN 1097-6 standard [30]. Natural fine limestone aggregate was used as fine aggregate.

Before mixing, and to avoid the loss of water from kneading by absorption, the LWAs were pre-saturated (reducing the uncertainty of lightweight aggregate water absorption during mixing [31]). During the mixing, the water content of the LWA and the surface water content were determined, to make the appropriate corrections and maintain a constant effective w/c ratio of 0.6.

## 3. Methodology

**Figure 1:**
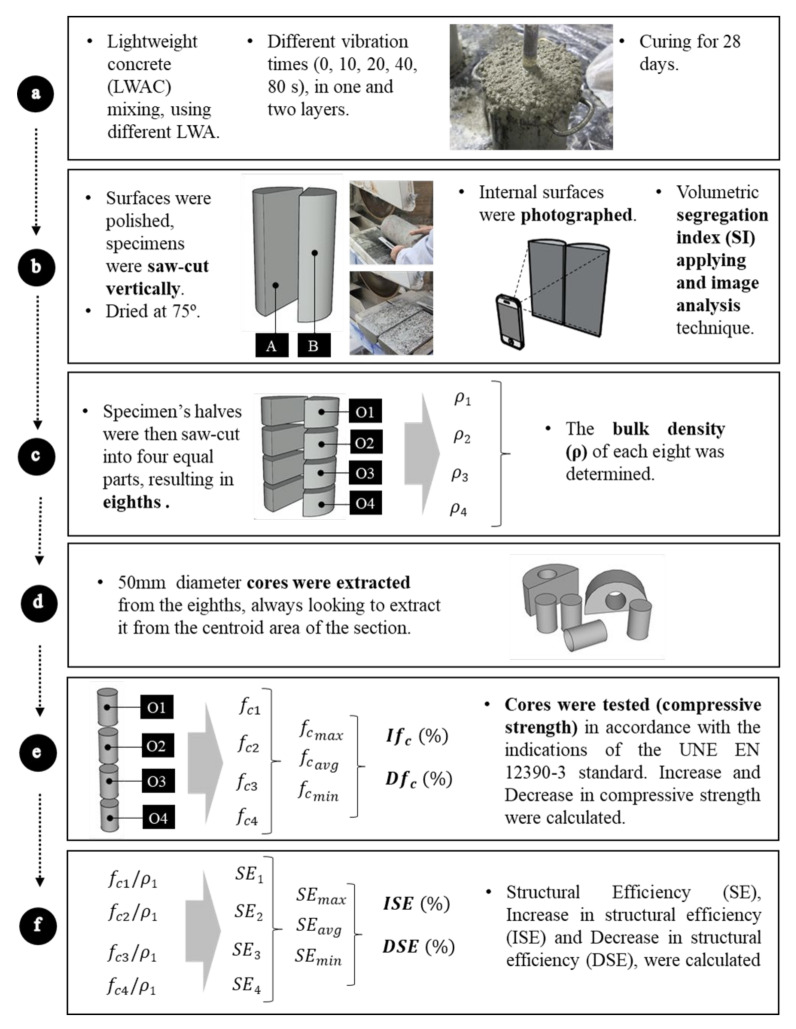
Methodology applied in this study. (**a**) Concrete production. (**b**) Samples were saw-cut, photographed and the segregation index was estimated. (**c**) Halves were saw-cut, densities were determined. (**d**) 50 mm cores were extracted. (**e**) Compressive strength tests were performed. (**f**) Structural Efficiency was determined.

The flowchart represented in Figure 1 shows the process followed in the research.

### 3.1. Manufacturing and Preparation of the Concrete Specimens

In the present study, the cylindrical samples (150 mm of diameter and 300 mm height) were compacted using an electric needle vibrator (AVMU, Enarco, Zaragoza, Spain) of 18,000 rpm/min and a 25 mm of diameter (Figure 1a). The specimens were vibrated with 5 different times (0, 10, 20, 40, 80 s), in one and two layers (Table 1). For each combination, four specimens were manufactured (20 specimens for each concrete mixture).

After being made and cured in the water at a temperature of 20 ± 1 °C for 28 days, the specimens were saw-cut through its longitudinal axis and their sections (identified as A and B) were photographed for image analysis (Figure 1b).

### 3.2. Photographing and Image Analyses Phase

**Figure 2:**
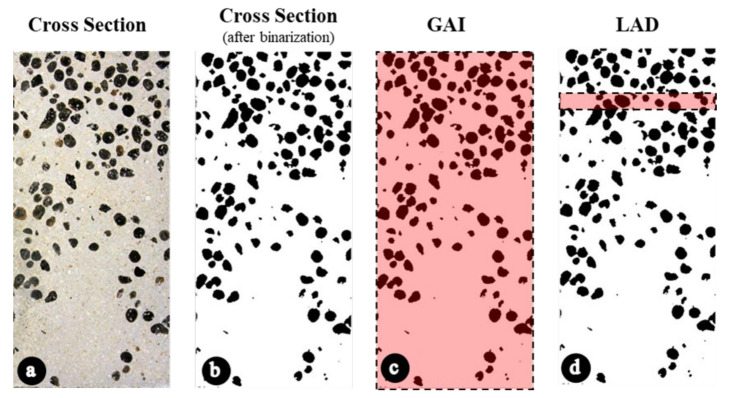
Information used to calculate the segregation index for each sample. (**a**) Cross section of sample. (**b**) Cross section of sample after binarization. (**c**) Data considered to calculate the volumetric fraction of aggregates (GAI) of each section. (**d**) Data considered to calculate the Local Absolute Difference (LAD) coefficients of each subsection (calculated at 701 subsections).

The two halves of each cylinder were photographed at the same time. The images of the sections were used to calculate the volumetric segregation index (SI) according Solak et al. [32] applying and image analysis technique. ImageJ, a freeware software, was used to process the images and to determine the black and white matrices. Adopting a point-counting technique, the data from these matrices was used to determine the volumetric fraction of aggregates (Global Aggregate Index - GAI) of each section (Figure 2b). Also, following the instructions of [32], each matrix was horizontally divided into 701 subsections, from which the local volumetric fractions of aggregate and mortar were obtained. The results of the local analyses were used to calculate the local absolute difference (LAD) coefficients (Figure 2d). The average of LADs resulted in the local distribution coefficient (LDC). Finally, the segregation index of Solak was calculated according to Equation (1).

(1) $$\mathrm{SI}\left(\%\right)=\frac{\mathrm{LDC}}{2\times \mathrm{GAI}\times \left(1-\mathrm{GAI}\right)}$$

The range of values of the SI varies from 0% (totally homogenous specimen) to 100% (totally segregated concrete where the aggregates are concentrated in the top of the specimen).

### 3.3. Compressive Strength Test

Subsequently the specimen’s halves were then saw-cut into four equal parts, resulting in eighths (identified as O1, O2, O3, and O4). To evaluate the variations caused by the segregation in the density (ρ) along the height of the specimens, the specimen’s halves were then saw-cut into four equal parts, resulting in eighths, which had their bulk densities (ρ) determined (Figure 1c).

To evaluate the variations caused by the segregation in the compressive strength (f_c_) along the height of the specimens, 50-mm diameter cores were extracted from the eighths always looking to extract it from the centroid area of the section (Figure 1d). Considering that the eighths have a transversal section with semi-circular geometry, the extraction of cores with circular section seemed more coherent.

The tests of compressive strength were carried out in accordance with the indications of the UNE EN 12390-3 standard [33], with attention to the specific indications of Annex B, where the procedure is recommended to test specimens with dimensions that do not meet the tolerances of the dimensions standardized in the UNE EN 12390-1 [34]. The cores extracted from the eighths have been tested in a compression testing machine of 20 kN, with loading speed of 0.25 MPa/s.

### 3.4. Compressive Strength Evaluation

To evaluate the influence of segregation on the compressive strength of concrete two indexes were established (Figure 1e):

Increase in compressive strength (${\mathrm{If}}_{c})$, calculated by Equation (2), and which represents the variation in compressive strength of one half of the sample, comparing the maximum strength found among the eighths of a half $\left({f_{c}}_{\max}\right)$ and the average strength of the eighths of the same half ($f_{c}{}_{\mathrm{avg}})$. Decrease in compressive strength (${\mathrm{Df}}_{c})$, calculated by Equation (3), which represents the variation in compressive strength of one half of the sample, comparing the minimum strength found between the eighths of a half ($f_{c}{}_{\min})$ and the average strength of the eighths of the same half ($f_{c}{}_{\mathrm{avg}})$.

(2) $${\mathrm{If}}_{c}\;\left(\%\right)=\;\frac{f_{c}{}_{max}}{f_{c}{}_{avg}}\times 100$$

(3) $$Df_{c}\;\left(\%\right)=\;\left|\frac{f_{c}{}_{min}}{f_{c}{}_{avg}}\;-\;1\right|\times 100$$

### 3.5. Structural Efficiency Evaluation

Other indexes, which indicates the structural efficiency of the material (Figure 1f) were also established:

Structural efficiency $\left(\mathrm{SE}\right)$, calculated by Equation (4), and which represents the proportion between the strength of a sample eighth $\left(f_{c}{}_{i}\right)$ in MPa and its density (ρi), in kg/m^3^.

Increase in structural efficiency (ISE), calculated by Equation (5), and which represents the variation in structural efficiency of one half of the sample, comparing the maximum structural efficiency found between the eighths of a half $\left({\mathrm{SE}}_{\max}\right)$ and the average structural efficiency of the eighths of the same half $({\mathrm{SE}}_{\mathrm{avg}}$).

Decrease in structural efficiency (DSE), calculated by Equation (6), which represents the variation in structural efficiency of one half of the sample, comparing the minimum structural efficiency found between the eighths of a half $\left({\mathrm{SE}}_{\min}\right)$ and the average structural efficiency of the eighths of the same half $({\mathrm{SE}}_{\mathrm{avg}}$).

(4) $$SE\;=\;\frac{f_{c}{}_{i}}{\rho i}$$

(5) $$ISE\;\left(\%\right)=\;\frac{SE_{max}}{SE_{avg}}\times 100$$

(6) $$DSE\;\left(\%\right)=\;\left|\frac{SE_{min}}{SE_{avg}}\;-\;1\right|\times 100$$

## 4. Results and Discussion

### 4.1. Compressive Strength

To analyze if the segregation of concrete directly affects the compressive strength, an ANOVA analysis complemented by the Tukey’s test (Honestly-significant-difference—HSD) has been carried out qualitatively evaluating this parameter. The segregation index, whose values are between 12% and 49%, were contrasted with the values of average compressive strength—average between the four cores extracted from each half ($f_{c}{}_{\mathrm{avg}}$)—of each sample. To analyze qualitatively, the data were grouped into categories: 10% ≥ SI > 20%; 20% ≥ SI >30%; 30% ≥ SI > 40% and 40% ≥ SI > 50%. In total 160 observations were made (two halves of 80 samples).

Given the R^2^, 10% of the variability of the dependent variable $f_{c}{}_{\mathrm{avg}}$ is explained by the explanatory variable (SI). Given the p-value of the F statistic computed in the ANOVA table (Table 3), and given the significance level of 5%, the information brought by the explanatory variable (SI) is significantly better than what a basic mean would bring, indicating that parameter SI affects the average compressive strength.

The Tukey’s test results have classified the four categories into two groups (A and B), indicating the existence of significant differences between them. As seen in Table 4, the behavior of the average compressive strength as a function of segregation seems to follow two different patterns, one for values below the category 20% to 30% and another to values above the category 20% to 30%.

**Figure 3:**
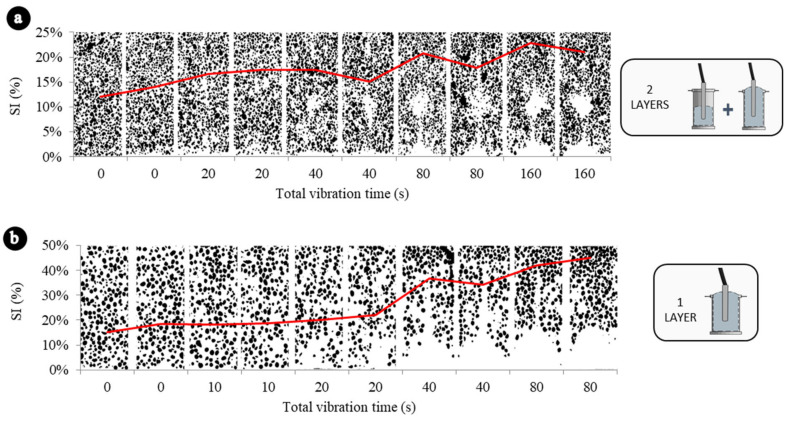
Examples of the evolution of segregation for two LWAC series. (**a**) Vibrated in two layers. (**b**) Vibrated in a single layer.

Figure 3. exemplifies two situations where a series of LWAC was vibrated in two layers (Figure 3a) and in one layer (Figure 3b). The graphics of all the series (16 in total) were included in the Supplementary Data.

**Figure 4:**
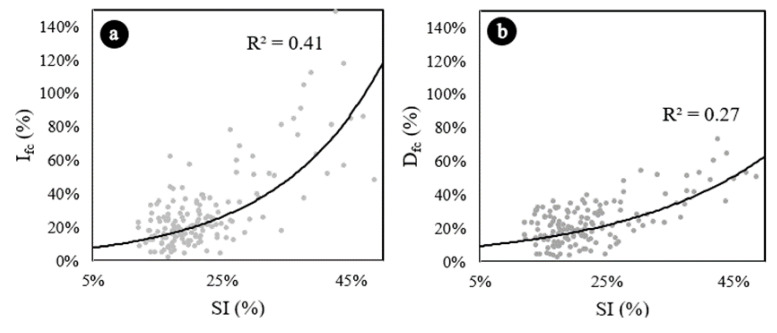
Exponential trend curves representing ${\mathrm{If}}_{c}$ vs SI and ${\mathrm{Df}}_{c}$ vs SI. (**a**) Increase in compressive strength due segregation. (**b**) Decrease in compressive strength due segregation. Exponential trend estimations.

**Figure 5:**
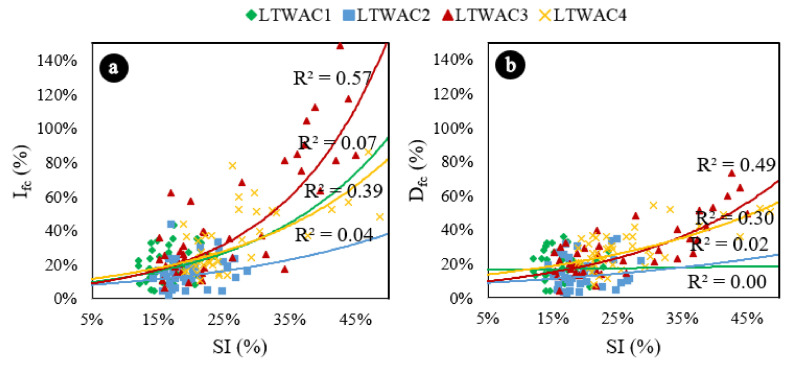
(**a**) Increase in compressive strength due segregation represented by concrete type. (**b**) Decrease in compressive strength due segregation represented by concrete type. Exponential trend estimations.

Figure 4a represents the variation of ${\mathrm{If}}_{c}$ as function of the segregation index. Results are consistent with the results of the ANOVA and the Tukey’s test. Concretes that have segregation indexes lower than 30% do not present any correlation between ${\mathrm{If}}_{c}$ and SI. However, starting at 30%, as SI increases, ${\mathrm{If}}_{c}$ increases. It probably occurs because at high values of SI the samples are clearly divided in two phases (mortar in the bottom and aggregates in the top), resulting in higher compressive strengths in those sections with mortar concentration. This analysis becomes more evident when the data are represented by concrete type (Figure 5a) and it is verified that the correlations of LWAC1 and LWAC2, which has presented SI lower than 30%, are not good: 0.07 and 0.034, respectively.

Concretes vibrated in two layers (LWAC1 and LWAC2) have presented less segregation, for vibration times even higher than the concretes vibrated in one layer, and because of their better homogeneity presented minor variations in the compressive strength of its sections. These results were also discussed in previous works [26].

From a safety point of view, the decrease in strength (sections that would contain an excessive amount of aggregates due to segregation) is more relevant than the increase in strength (sections that would contain an excessive amount of mortar), once those are mostly the areas where the concrete failure begins [35]. Figure 4b represents the variation of ${\mathrm{Df}}_{c}$ as function of the SI.

The results are also consistent with ANOVA and Tukey’s test results. Concretes with SI lower than 25% do not present any correlation between ${\mathrm{Df}}_{c}$ and SI. However, starting at 25%, as SI increases, ${\mathrm{Df}}_{c}$ increases, probably because at high values of SI, the samples are clearly divided in two phases (mortar in the bottom and aggregates in the top), resulting in lower compressive strengths in those sections with aggregate concentration. In the same way, this analysis becomes more evident when the data are represented by concrete type (Figure 5b) and it is verified that the correlations of LWAC1 and LWAC2, which has presented SIs lower than 25%, are very poor: 0.00 and 0.02, respectively. The volumetric fraction of aggregates—related to the theoretical densities in this paper—also affected the reduction of compressive strength due segregation. Concretes manufactured in one layer, and with density of 1700 kg/m^3^ presented higher reductions of compressive strength due segregation than concretes with theoretical densities of 1900 kg/m^3^ (one layer).

### 4.2. Position of the Critical Section

**Figure 6:**
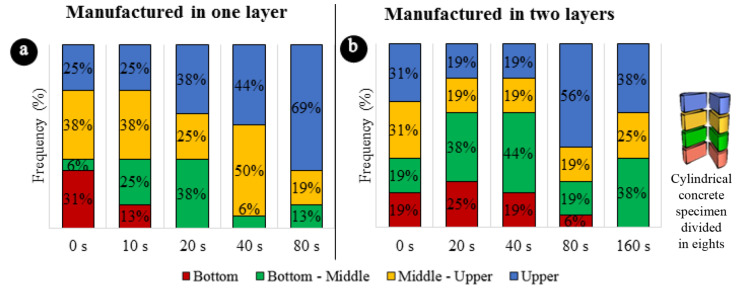
Frequency of the critical section in each eighth. (**a**) Concretes manufactured in one layer. (**b**) Concretes manufactured in two layers.

Figure 6 shows the position of the critical section (eighth with $f_{c}{}_{\min}$) as a function of the vibration time. Figure 6b shows the frequency of the critical section in each eighth, for each vibration time, for the concrete manufactured in two layers. With the increase in vibration energy, the critical section became more frequent in the middle and upper eighths, until at the maximum vibration energy (t = 160 s), when no cases of critical sections were found in the lower eighth (red).

In concretes vibrated in one layer, the displacement of the critical section towards the upper eighths is even more evident. In Figure 6a it is observed that, in short vibration times, the frequency of the critical section is well distributed in the eighths, and with the increase of the vibration time, the upper eighth (blue) gradually registered more cases of critical section, until in the maximum time (t=80s), in 69% of the cases the critical section is in the upper eighth.

### 4.3. Structural Efficiency

**Figure 7:**
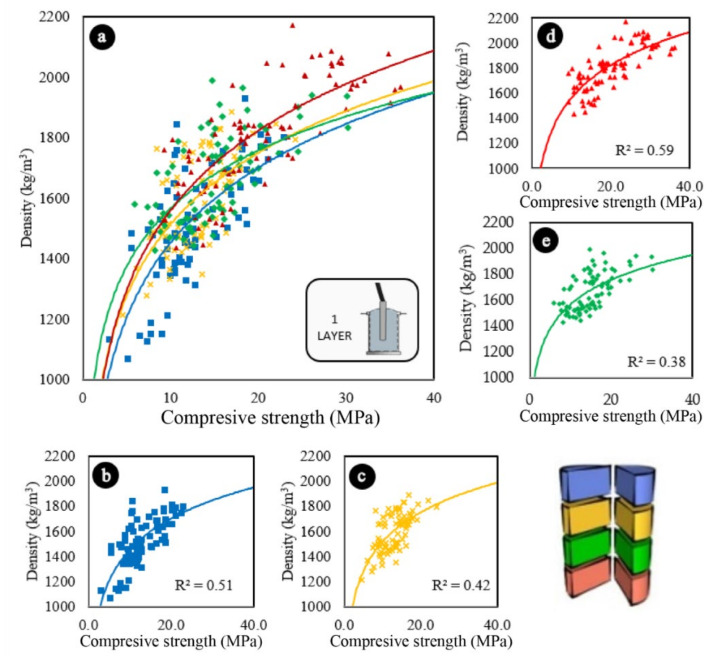
Variations in the compressive strength of samples vibrated in one layer according to their densities (**a**) All sections. (**b**) Upper sections. (**c**) Middle-upper sections. (**d**) Bottom sections. (**e**) Bottom-middle sections. Logarithmic trend estimations.

**Figure 8:**
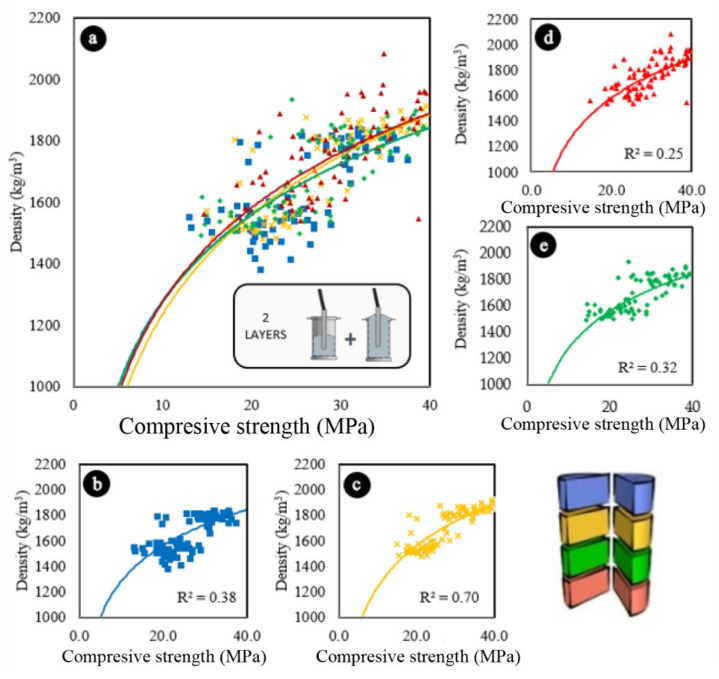
Variations in the compressive strength of samples vibrated in two layers according to their densities. (**a**) All sections. (**b**) Upper sections. (**c**) Middle-upper sections. (**d**) Bottom sections. (**e**) Bottom-middle sections. Logarithmic trend estimations.

In general, the compaction in two layers led to a reduction in the overall density of the samples, most likely because the trapped air was removed more effectively in these cases. This can be seen when comparing the graphs in Figure 7a, Figure 8a.

Two-layer compaction also affected the compressive strength results. When comparing the graph in Figure 7a (vibration in one layer) with the graph in Figure 8a (vibration in two layers), it can be seen that all the curves moved to the right, so that the concretes vibrated in two layers presented superior compressive strength results.

Although the trends and the results of R^2^ vary depending on the position of each eighth within the samples, in both one-layer and two-layer vibrating concretes, it can be seen through Figure 7 and Figure 8 that the denser, the greater in terms of compressive strength.

Concretes compacted in one layer have been shown to be more sensitive to vibration than concretes vibrated in two layers, especially in the upper region of the samples. When comparing Figure 7b with Figure 8b, and Figure 7c with Figure 8c, it can be seen that the curves of Figure 7b,c show more vertical trends than the curves of 8b and 8c, indicating that the concretes vibrated in one layer showed a greater loss of resistance of its upper sections due to the increase in the concentration of lightweight aggregates due to segregation, which resulted in a consequent reduction in density.

To analyze if the segregation of concrete directly affects structural efficiency, the data were also analyzed using an ANOVA complemented by a Tukey test (HSD). The segregation results were contrasted with the values of average structural efficiency—average between the results of four cores extracted from each half (${\mathrm{SE}}_{\mathrm{avg}})$—of each sample. In the same way that the compressive strength analysis was performed, the data were grouped into categories: 10% ≥ S > 20%; 20% ≥ SI > 30%; 30% ≥ SI > 40% and 40% ≥ SI > 50%. In total, 160 observations were made (two halves of 80 samples).

Given the R^2^, 10% of the variability of the dependent variable ${\mathrm{SE}}_{\mathrm{avg}}$ is explained by the explanatory variable (SI). Given the p-value of the F statistic computed in the ANOVA table (Table 5), and given the significance level of 5%, the information brought by the explanatory variable (SI) is significantly better than what a basic mean would bring, indicating that parameter SI average structural efficiency.

The Tukey’s test results have also classified the four categories into two groups (A and B), indicating the existence of significant differences between them. As seen in Table 6, the behavior of the average structural efficiency as a function of segregation seems to follow two different patterns, one for values below the category 20% to 30% and another to values above the category 20% to 30%.

**Figure 9:**
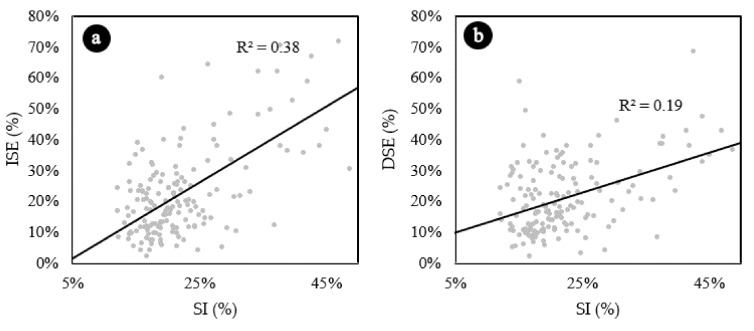
(**a**) Increase in structural efficiency (ISE) due segregation. (**b**) Decrease in structural efficiency (DSE) due segregation. Linear trend estimations.

**Figure 10:**
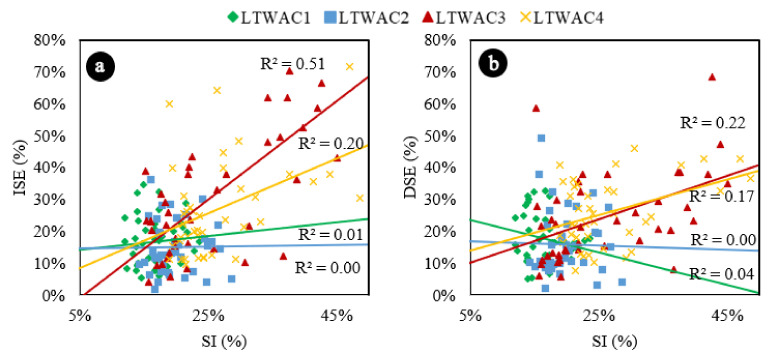
(**a**) Increase in structural efficiency due segregation represented by concrete type. (**b**) Decrease in structural efficiency due segregation represented by concrete type. Linear trend estimations.

Figure 9a represents the variation of ISE as function of the segregation index. Results are consistent with the results of the ANOVA and the Tukey’s test. Concretes that have segregation indexes lower than 30% do not present any correlation between ISE and SI. However, starting at 30%, as SI increases, ISE increases. This analysis becomes more evident when the data are represented by concrete type (Figure 10a) and it is verified that the correlations of LWAC1 and LWAC2, which has presented SI lower than 30%, are not good: 0.01 and 0.00, respectively.

Like the results obtained by Solak et al. [26], concretes vibrated in two layers (LWAC1 and LWAC2) have presented less segregation, for vibration times even higher than the concretes vibrated in one layer, and because of their better homogeneity presented minor variations in the compressive strength, density, and as result, minor variations of structural efficiency on its sections.

Figure 9b represents the variation of DSE as function of the SI. Concretes with SI lower than 30% do not present any correlation between DSE and SI. However, starting at 30%, as SI increases, DSE increases. Similar to the previously analyzed parameter, this analysis becomes more evident when the data are represented by concrete type (Figure 10b) and it is verified that the correlations of LWAC1 and LWAC2, which presented SIs lower than 30%, are very poor: 0.0006 and 0.0404, respectively.

**Figure 11:**
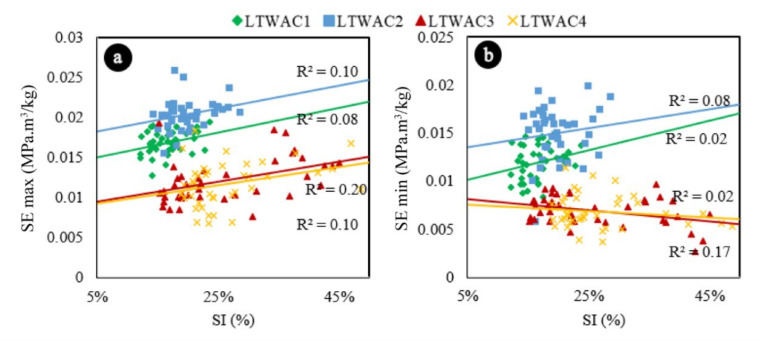
(**a**) Maximum structural efficiency (${\mathrm{SE}}_{\max}$) versus segregation, represented by concrete type. (**b**) Minimum structural efficiency (${\mathrm{SE}}_{\min}$) versus segregation, represented by concrete type. Linear trend estimations.

For all structural efficiency parameters analysed (${\mathrm{SE}}_{\max}$, ${\mathrm{SE}}_{\min},$ and ${\mathrm{SE}}_{\mathrm{avg}}$), the concretes that were vibrated in two layers (LWAC 1 and LWAC2, green and blue in the Figure 11), presented higher values when compared to the concretes that were vibrated in one layer (LWAC 3 and LWAC 4, yellow and red in the Figure 11). It is not possible to state that the segregation caused significant impacts on the structural efficiency of the materials, since no clear trends were found for the four studied concretes. This is verified through the linear and approximately horizontal aspects of the data distributions, as well as through the low values of R^2^.

## 5. Conclusions

This paper presents an experimental study on lightweight aggregate concretes (LWAC) with an analysis of variations in the density, compressive strength, and structural efficiency of samples affected by segregation caused by excessive vibration. From the results presented in this study, the following conclusions are drawn:

- The manufacturing of the samples in two layers ensured a homogeneity of the mixture inside of the samples and consequently avoided an excessive reduction of compressive strength.
- The decrease in the compressive strength was observed with more evidence from segregation indexes above 25%. Other compressive strength tests considering specimens with standard dimensions instead of the cores extracted from the samples could explain this phenomenon more precisely.
- Concretes manufactured in one layer presented higher reductions of compressive strength due to segregation than concretes manufactured in two layers.
- For all structural efficiency parameters analysed (${\mathrm{SE}}_{\max}$, ${\mathrm{SE}}_{\min}$ and ${\mathrm{SE}}_{\mathrm{avg}}$), the concretes that were vibrated in two layers presented higher values when compared to the concretes that were vibrated in one layer.

Although several parameters that affect segregation in LWAC have been evaluated in this article, other factors still need to be studied in future research. This article could be complemented with compression strength tests using conventional size samples, LWA with other sizes, and concretes with other dosages.

## Figures and Tables

**Table 1:** Manufacturing characteristics and mix proportions to produce 1 m^3^ of concrete. Adapted table from [26].

Concrete Identification	Type of LWA	Amount of Fine Aggregate in a Cubic Meter (kg/m^3^)	Amount of LWA in a Cubic Meter (kg/m^3^)
LWAC1	HS	723.9	416.2
LWAC2	HS	1046.0	294.0
LWAC3	M	991.1	148.9
LWAC4	M	1234.8	105.2

**Table 2:** Characteristics of aggregates and the methods/standards used for testing. Adapted table from [26].

Property	Method	Arlita Leca M	Arlita Leca HS	Fine Aggregate
Apparent particle density (kg/ m^3^)	According to Ref. [27]	482	1019	2688
Bulk density (kg/ m^3^)	UNE EN 1097-3 [28]	269	610	1610
Water absorption (%)	According to Ref. [27]	36.6	12.2	0.12
Particle size (di/Di)	UNE EN 933-1 [29]	6/10	4/10	0/4
Crushing strength (MPa)	According the manufacturer	1.0	5.0	-

**Table 3:** Results of ANOVA. DF (degrees of freedom), F (F ratio) and Pr (p-value).

Source	DF	Sum of Squares	Mean Squares	F	Pr > F
Model	3	917.26	305.76	5.53	0.001
Error	156	8626.39	55.30	-	-
Corrected Total	159	9543.64	-	-	-

Computed against model Y = Mean(Y).

**Table 4:** Tukey’s test results. LS (Least-squares).

Category	LS Means	Standard Error	Lower Bound (95%)	Upper Bound (95%)	Groups	
10% ≥ SI >20%	23.34	0.84	21.67	24.99	A	
20% ≥ SI >30%	21.17	0.98	19.24	23.09	A	B
30% ≥ SI >40%	16.33	1.92	12.53	20.12		B
40% ≥ SI >50%	15.99	2.63	10.80	21.19		B

**Table 5:** Results of ANOVA. DF (degrees of freedom), F (F ratio) and Pr (p-value).

Source	DF	Sum of Squares	Mean Squares	F	Pr > F
Model	3	0.0004	0.0001	10.1978	0.0001
Error	156	0.0021	0.0000	-	-
Corrected Total	159	0.0026	-	-	-

Computed against model Y=Mean(Y).

**Table 6:** Tukey’s test results. LS (Least-squares).

Category	LS Means	Standard Error	Lower Bound (95%)	Upper Bound (95%)	Groups	
10% ≥ SI >20%	0.014	0.000	0.013	0.015	A	
20% ≥ SI >30%	0.012	0.000	0.011	0.013		B
30% ≥ SI >40%	0.010	0.001	0.008	0.012		B
40% ≥ SI >50%	0.009	0.001	0.006	0.011		B

## Supplementary Materials

The following are available online at https://www.mdpi.com/1996-1944/13/24/5754/s1, Figure S1: Segregation index for each sample according to the vibration time applied. LWAC1; Figure S2: Density of the eighths of sample, according to the time of vibration applied. LWAC1; Figure S3: Segregation index for each sample according to the vibration time applied. LWAC2; Figure S4: Density of the eighths of sample, according to the time of vibration applied. LWAC2; Figure S5: Segregation index for each sample according to the vibration time applied. LWAC3; Figure S6: Density of the eighths of sample, according to the time of vibration applied. LWAC3; Figure S7: Segregation index for each sample according to the vibration time applied. LWAC4; Figure S8: Density of the eighths of sample, according to the time of vibration applied. LWAC4.
