# Supplementary material for: Influence of The Segregation Phenomenon on Structural Efficiency of Lightweight Aggregate Concretes
Source: Materials (Basel). 2020 Dec 16;13(24):5754. doi: 10.3390/ma13245754 (PMC7766865; doi:10.3390/ma13245754)
Supplement: Supplementary file 1 [file materials-13-05754-s001.pdf]

Supplementary Materials:

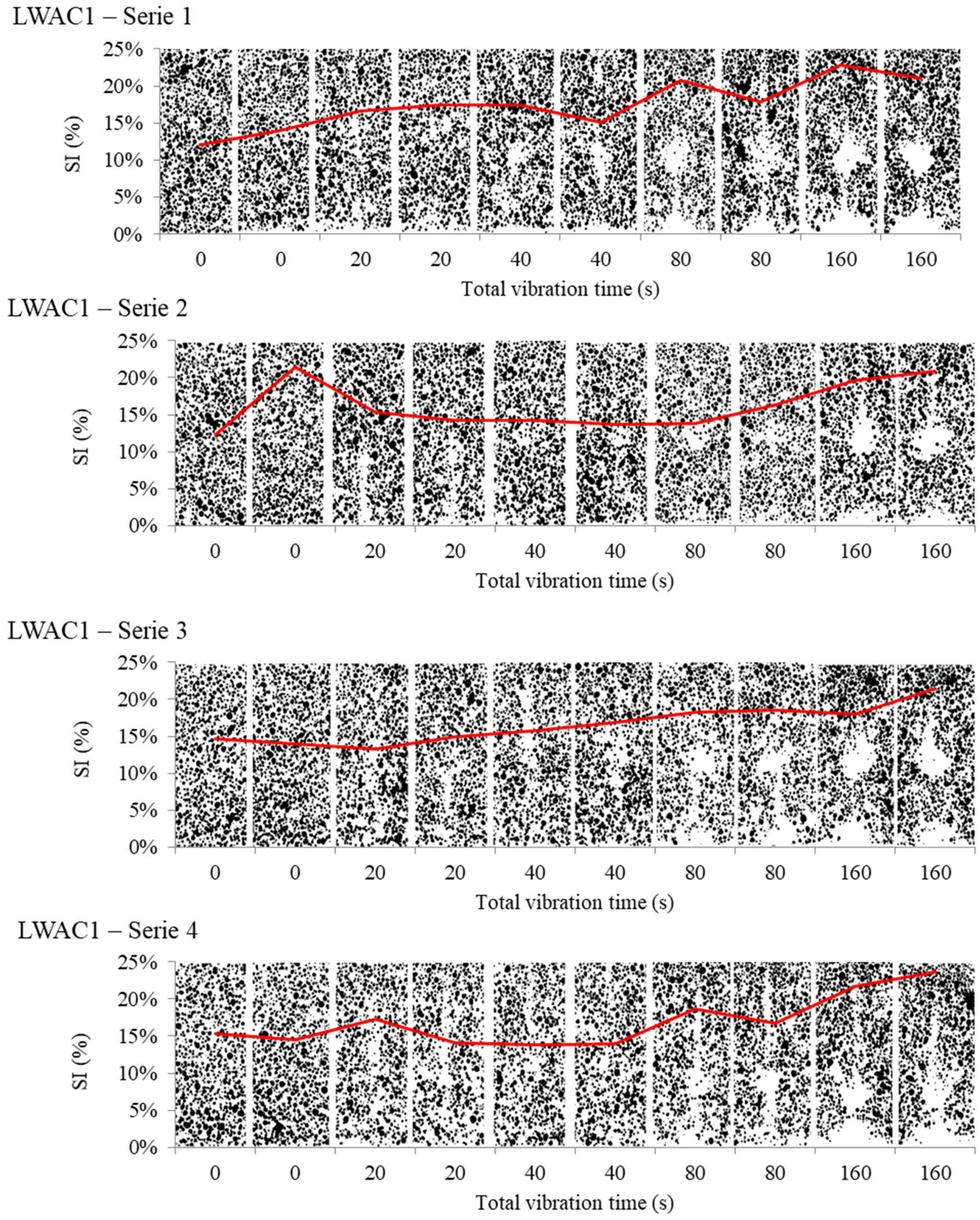

Figure S1. Segregation index for each sample according to the vibration time applied. LWAC1.

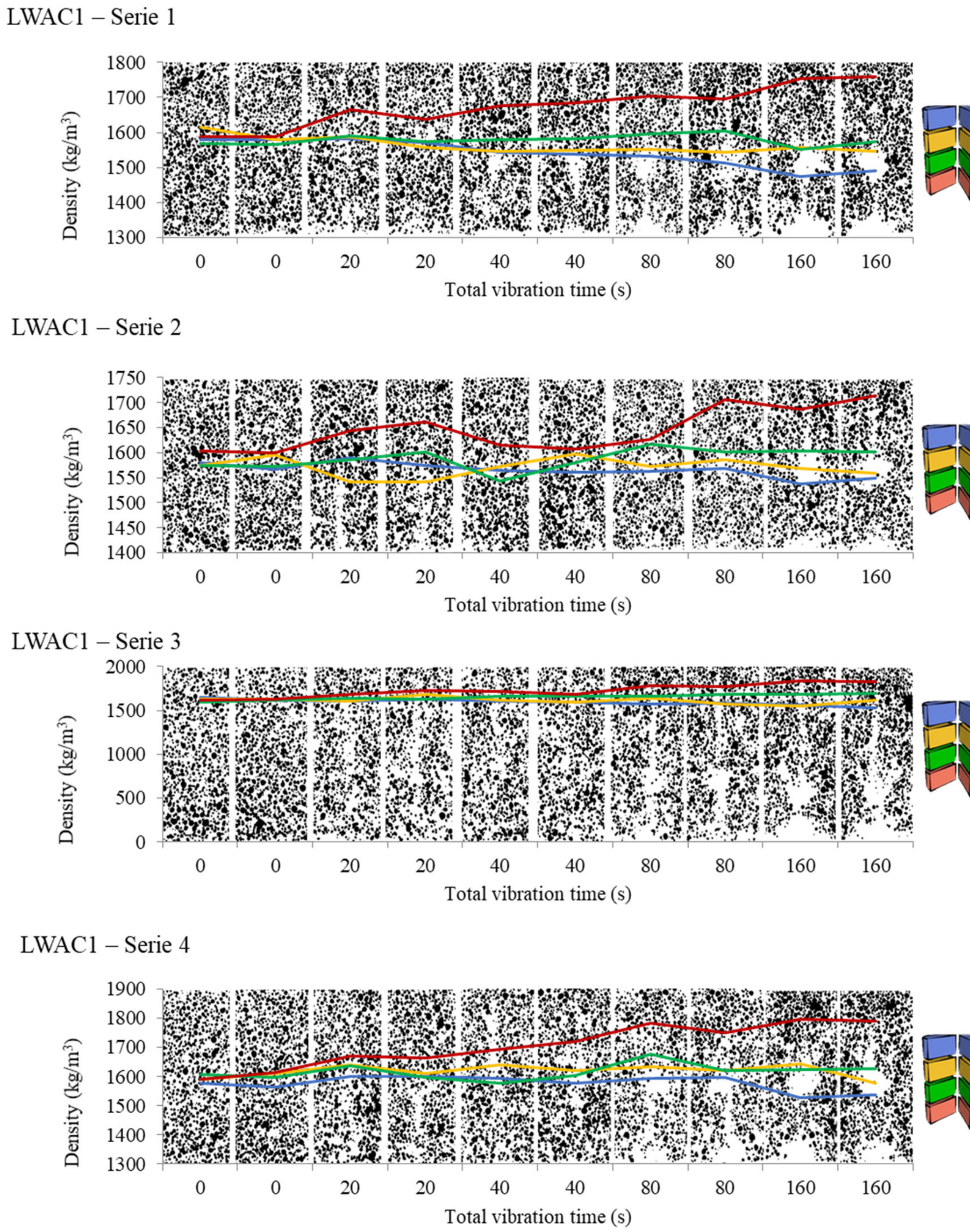

Figure S2. Density of the eighths of sample, according to the time of vibration applied. LWAC1.

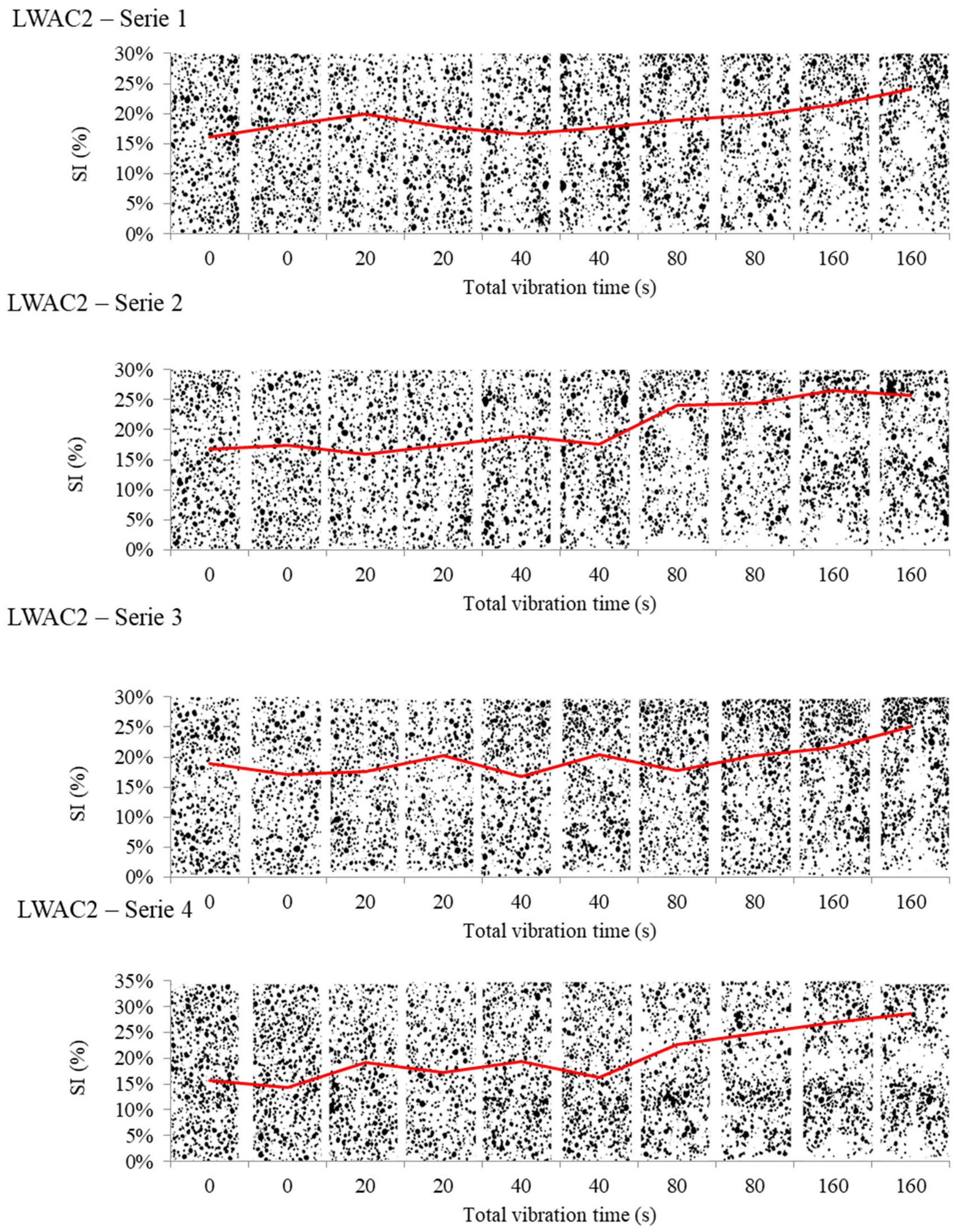

Figure S3. Segregation index for each sample according to the vibration time applied. LWAC2.

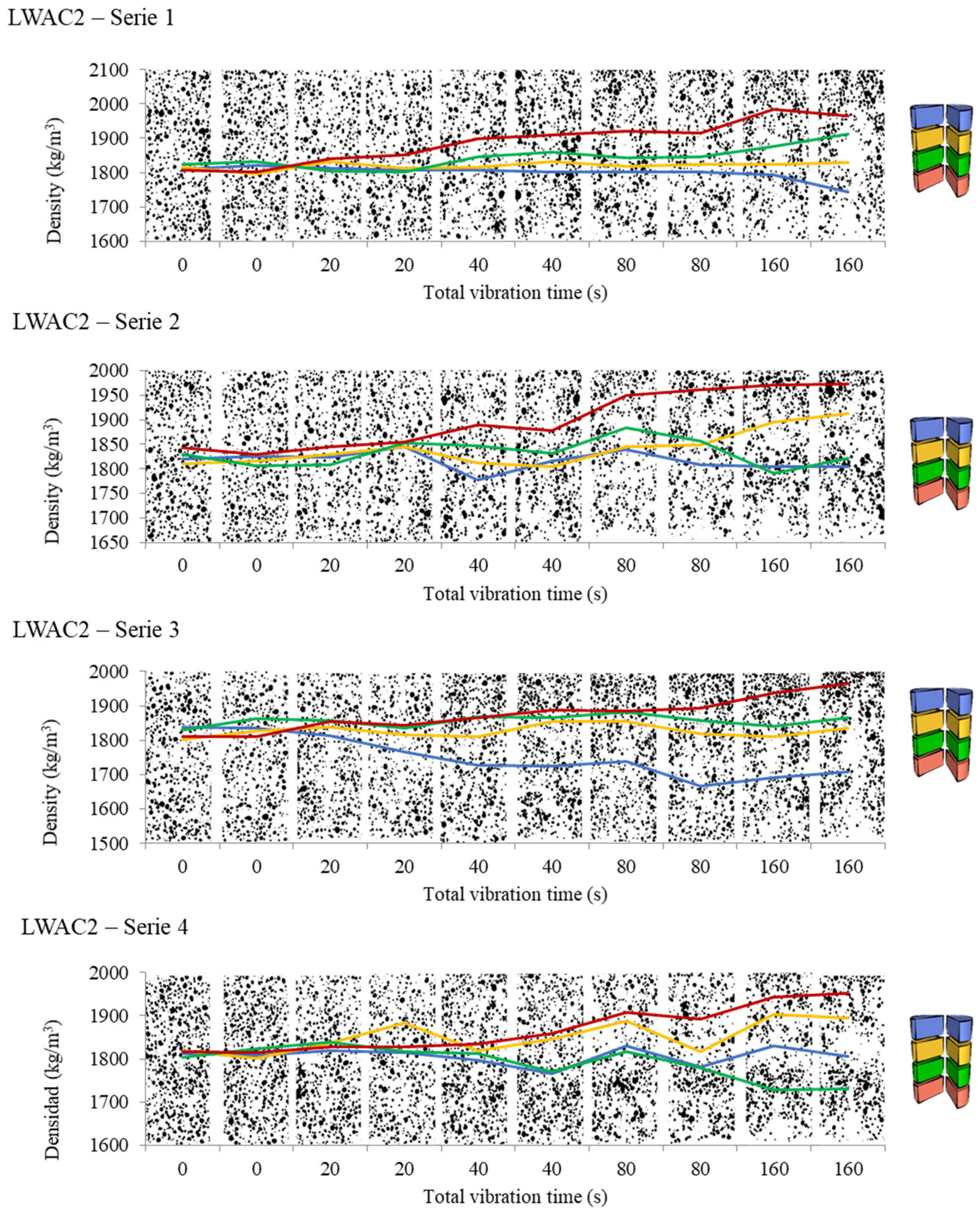

Figure S4. Density of the eighths of sample, according to the time of vibration applied. LWAC2.

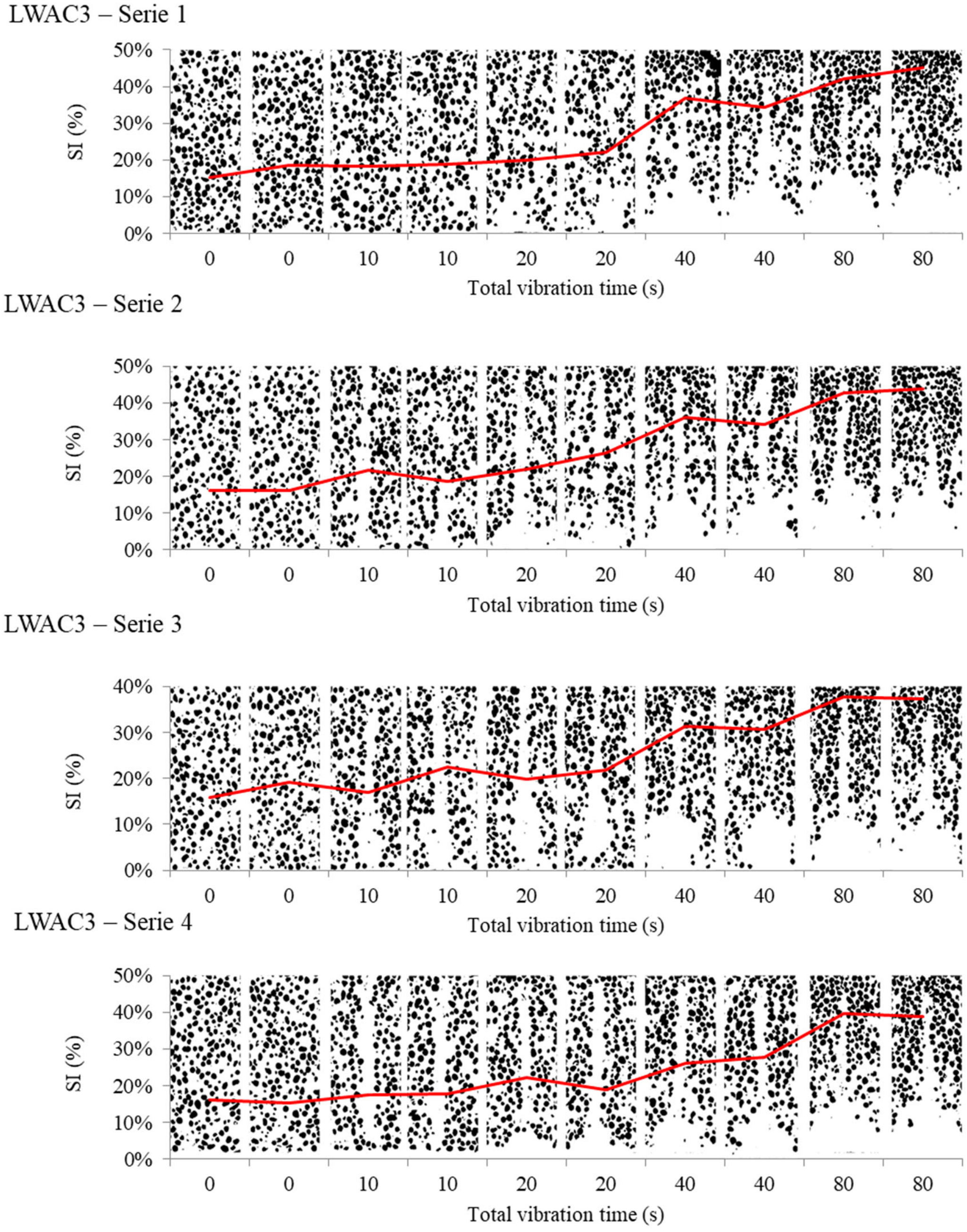

Figure S5. Segregation index for each sample according to the vibration time applied. LWAC3.

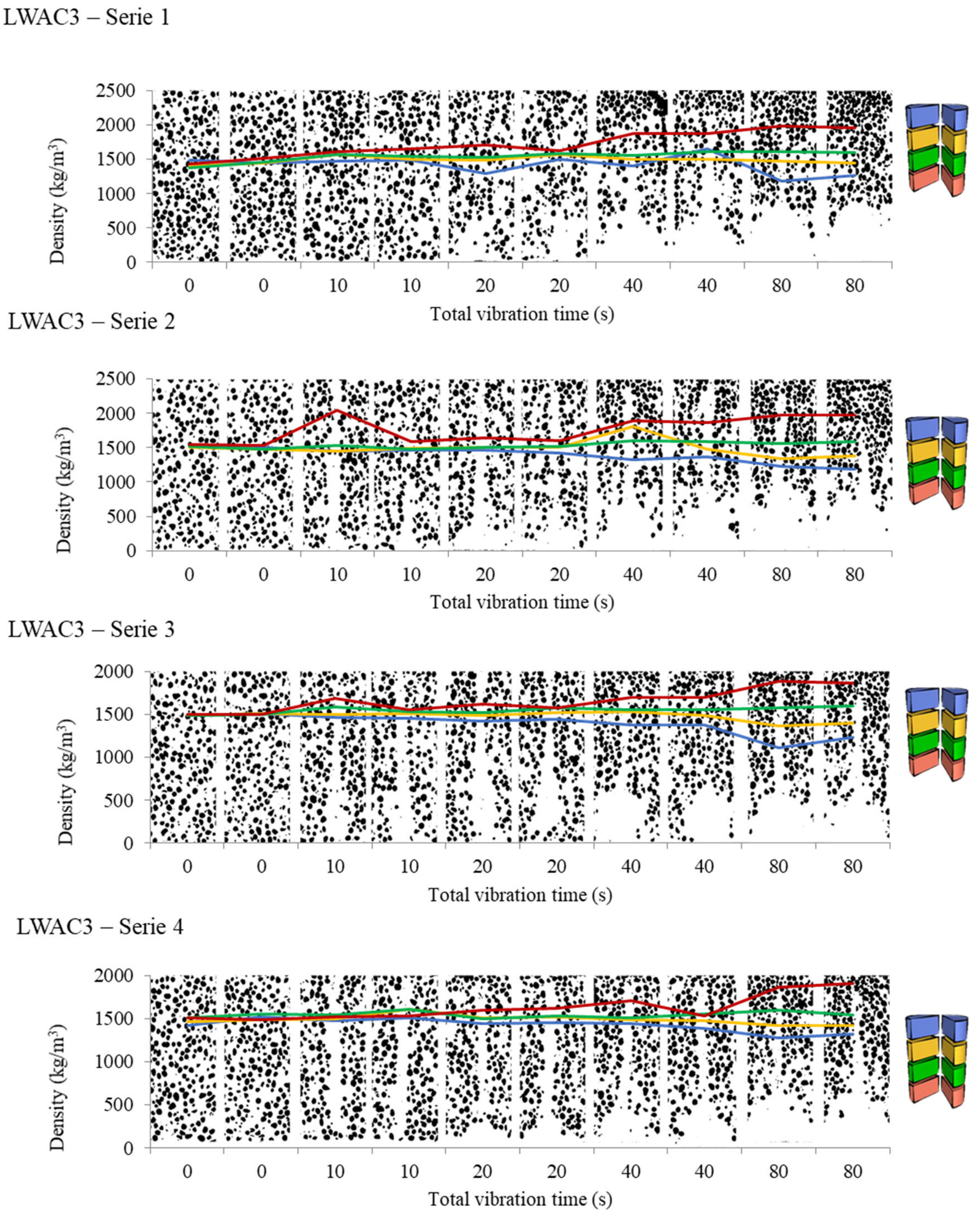

Figure S6. Density of the eighths of sample, according to the time of vibration applied. LWAC3.

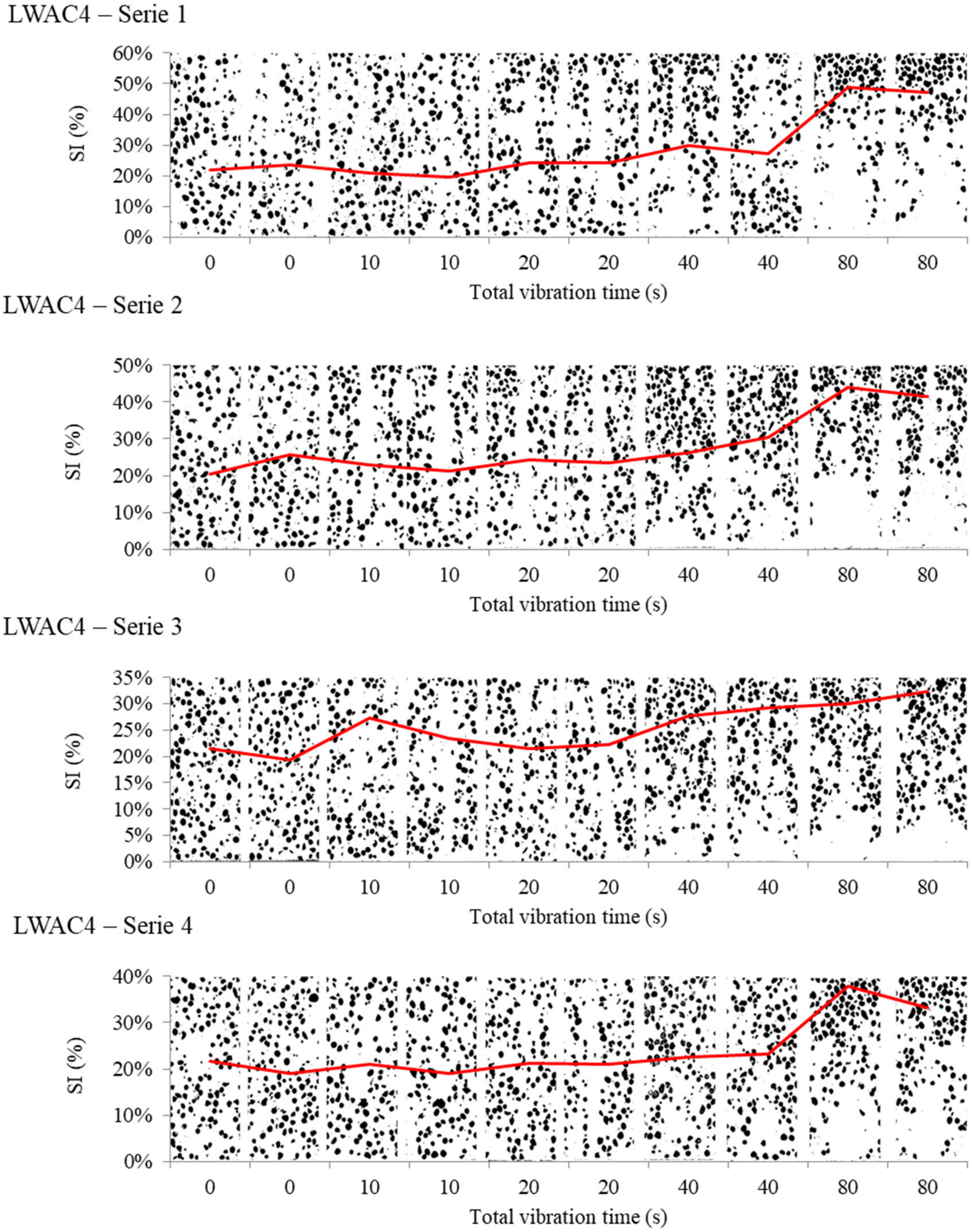

Figure S7. Segregation index for each sample according to the vibration time applied. LWAC4.

LWAC4 – Serie 1

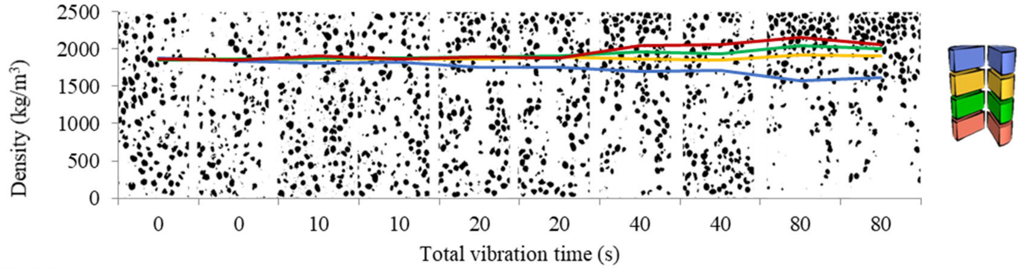

LWAC4 – Serie 2

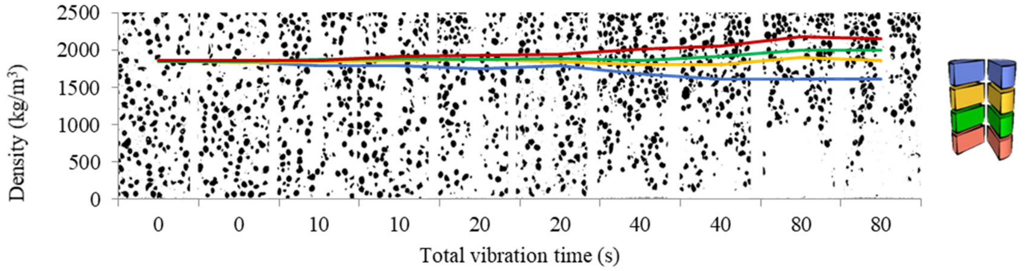

LWAC4 – Serie 3

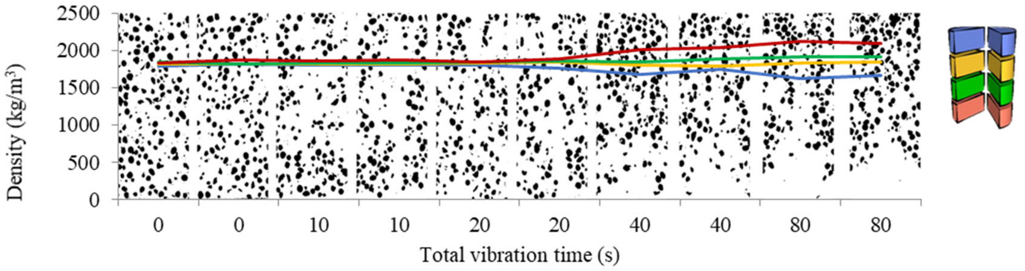

LWAC4 – Serie 4

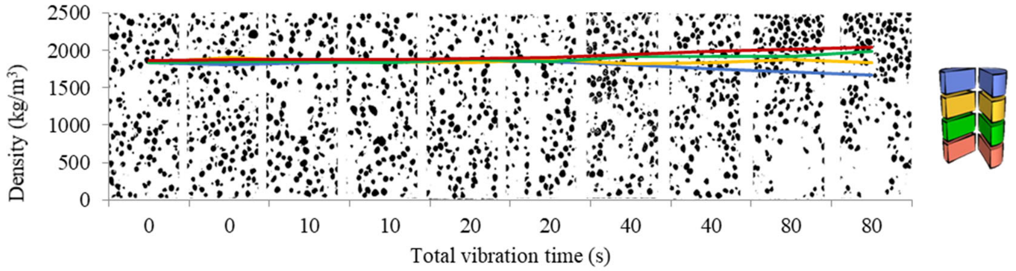

Figure S8. Density of the eighths of sample, according to the time of vibration applied. LWAC4.
